# SMURF: soft-segmentation for single-cell reconstruction and topological analysis of spatial transcriptomic data

**DOI:** 10.1101/2025.05.28.656357

**Published:** 2025-11-01

**Authors:** Juanru Guo, Simona Sarafinovska, Ryan A. Hagenson, Mark C. Valentine, David Y. Chen, William H. McCoy, Joseph D. Dougherty, Robi D. Mitra, Brian D. Muegge

## Abstract

High-resolution spatial transcriptomics requires new computational methods to accurately assign transcripts to individual cells. We developed SMURF (Segmentation and Manifold UnRolling Framework), a novel, cross-platform, soft-segmentation algorithm that maps mRNAs from barcoded capture spots to nearby nuclei. SMURF also “unrolls” complex tissue architectures by projecting cells onto Cartesian coordinates, enabling analysis of cell-type organization and gene-expression gradients in intact tissues. We benchmarked SMURF and found it assigns mRNAs to single cells more accurately than existing approaches. We evaluated SMURF’s ability to unroll complex tissues at cell-type resolution across multiple tissues and platforms. This analysis revealed previously unrecognized zonation of gene-expression programs, identified the transcription factors that regulate these patterns, and provided evidence that regional gene expression at the intestinal tip is reprogrammed by luminal environmental signals. Together, these results establish SMURF as a powerful framework for analyzing gene expression of cells within their native tissue contexts.

## Full Text Availability

The license terms selected by the author(s) for this preprint version do not permit archiving in PMC. The full text is available from the preprint server.

